# Causal Relationship Between Neurotrophins and Normal Pressure Hydrocephalus: Insights From a Bidirectional Two‐Sample Mendelian Randomization Study

**DOI:** 10.1002/brb3.71309

**Published:** 2026-03-30

**Authors:** Tao Xu, Qiang Gan, Handong Wang, Haifeng Liu, Xiwen Huang

**Affiliations:** ^1^ Department of Neurosurgery Nanjing BenQ Medical Center Nanjing China

**Keywords:** ciliary neurotrophic factor levels (CNTF), instrumental variables, Mendelian randomization, neurotrophins, normal pressure hydrocephalus (NPH)

## Abstract

**Introduction:**

Researchers have postulated a link between higher levels of brain‐derived neurotrophic factor (BDNF) and more favorable outcomes in patients with normal pressure hydrocephalus (NPH). However, there is no clear evidence regarding the causal association between neurotrophins and NPH. To delve deeper into this potential connection, scientists employed a rigorous method known as bidirectional Mendelian randomization (MR). This technique was utilized to explore the causal impact of various neurotrophins—such as BDNF, nerve growth factor (NGF), neurotrophin‐3 (NT‐3), NT‐4, ciliary neurotrophic factor (CNTF), and glial cell line‐derived neurotrophic factor (GDNF)—on the development or progression of NPH.

**Methods:**

To investigate the causal relationship between five neurotrophin subtypes and NPH, we designed a two‐sample Mendelian randomization (MR) study using comprehensive genome‐wide association study (GWAS) data. Our primary approach involved the inverse‐variance weighted (IVW) method. We also conducted reverse causality analysis to ensure robustness. Furthermore, we implemented complementary methods like the weighted median (WM), weighted mode, and MR‐Egger to strengthen our findings. Sensitivity analyses, including MR‐Egger, MR‐PRESSO, leave‐one‐out, and Cochran's Q tests, were employed to validate results, explore heterogeneity and pleiotropy, and pinpoint potential biases.

**Results:**

MR analysis of genetic prediction showed no statistical association of neurotrophins on NPH. However, a reverse analysis indicated a causal association between NPH and two neurotrophins: CNTF and GDNF. Specifically, individuals with NPH had a lower risk of CNTF (odds ratio: 0.7963, with a 95% confidence interval of 0.6537 to 0.9701, *p* = 0.0237) and a slightly reduced risk of GDNF (odds ratio: 0.9576, with a 95% confidence interval of 0.9226 to 0.9940, *p* = 0.0230). MR‐Egger regression showed that pleiotropy did not affect the analysis. In addition, MR‐PRESSO detected no outliers, and a leave‐one‐out analysis verified the robustness of the results.

**Conclusion:**

NPH was negatively and causally associated with CNTF and GDNF. Additional research is crucial to uncover the underlying mechanisms and devise strategies, including nutritional guidelines, to prevent NPH.

## Graphical abstract

This Mendelian randomization study reveals that normal pressure hydrocephalus (NPH) causally leads to lower levels of the neurotrophins CNTF and GDNF. This finding provides new genetic evidence for the role of neurotrophic factors in NPH, suggesting potential avenues for further research into prevention and treatment strategies.

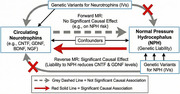

**Abbreviations**
BDNF: brain‐derived neurotrophic factor
CNTF: ciliary neurotrophic factor
GDNF: glial cell line‐derived neurotrophic factor
GWAS: genome‐wide association study
IVW: inverse‐variance weighted
MR: mendelian randomization
NGF: nerve growth factor
NPH: normal pressure hydrocephalus
WM: weighted median

## 1 Introduction

Normal pressure hydrocephalus (NPH) is characterized by a distinctive set of symptoms that primarily affect older adults (Jaraj et al. 2014). This clinical syndrome encompasses cognitive decline, gait instability, and urinary incontinence, often accompanied by neuroimaging evidence of enlarged ventricles without elevated cerebrospinal fluid pressure (Adams et al. 1965). A Swedish cross‐sectional study revealed a prevalence of 1.5% among individuals aged 70 and older, with an overall imaging‐based prevalence of 5.1%. Given the aging population, the incidence of NPH has been rising steadily (Constantinescu et al. 2024). Traditionally, ventriculoperitoneal shunt surgery has been the primary treatment option, though it is associated with notable postoperative complications (Israelsson et al. 2020). Unfortunately, the exact causes and pathophysiological mechanisms underlying NPH remain largely unknown, posing challenges for both diagnosis and management.

Neurotrophic factor is one of the components of the cerebrospinal fluid circulatory system that feeds the neuronal network, and intervening in it may be potentially relevant for the treatment of hydrocephalus (Johanson et al. 2005). The neurotrophin family encompasses a diverse array of six identified members, each with unique functions and binding specificities. These include glial cell line‐derived neurotrophic factor (GDNF), brain‐derived neurotrophic factor (BDNF), neurotrophin‐3 (NT‐3), NT‐4, ciliary neurotrophic factor (CNTF), and nerve growth factor (NGF). These neurotrophins exhibit specific interactions with the Trk family of receptors. These neurotrophins exhibit specific interactions with the Trk family of receptors. For instance, NGF selectively binds to TrkA, while BDNF and NT‐4/5 preferentially bind to TrkB. NT‐3, on the other hand, demonstrates an affinity for TrkC while maintaining the ability to interact with both TrkA and TrkB (Weihrauch et al. 2023). A basic study concluded that NT‐4 may improve NPH after brain injury by targeting TrkB (Wang et al. 2020). CNTF is a neuroprotective agent in the retina, which has significant and widespread neuroprotective effects on retinal neurons (Do Rhee et al. 2022). Its protective prowess extends to other neurons, influencing neurogenesis, regeneration, and survival (Guo et al. 2022). GDNF, a member of the transforming growth factor beta superfamily, was thought to be closely related to neuronal function and brain diseases (Shpak et al. 2023; Tao et al. 2019). One case‐control study reported significantly lower serum BDNF levels in NPH patients compared to healthy controls (Laske et al. 2007). However, contrasting findings from another study by Med et al. indicated no significant reduction in BDNF serum concentrations among NPH patients (Mehmedika‐Suljić et al. 2023). In addition, studies of cerebrospinal fluid have shown elevated NGF and NT‐3 concentrations in children with NPH (Hochhaus et al. 2001). However, to date, there is a lack of research on the relationship between neurotrophins and NPH. The potential causal link between genetic susceptibility to neurotrophins and the risk of developing NPH remains elusive. Further research is imperative to elucidate the precise mechanisms underlying these interactions and to explore the therapeutic potential of neurotrophins in the management of hydrocephalus.

In this study, we have adopted the sophisticated statistical technique of Mendelian randomization (MR) to explore the potential causal links between various exposures and health outcomes (Birney 2022). MR‐based study designs allow for the investigation of many exposures that are not amenable to randomized controlled trial (RCT) studies (Guo et al. 2021). Recent advancements have seen MR being increasingly utilized to elucidate causal mechanisms underlying NPH, facilitated by the expanding availability of extensive genomic resources, including large‐scale GWASs and the UK Biobank. A notable example is the study by Yuze et al., which identified PD as a risk factor for NPH using MR, highlighting its potential to uncover novel disease associations (Song et al. 2024). In addition, Ziang et al. concluded that primary hypertension is a pathogenic risk factor for NPH (Deng et al. 2023). Despite the growing interest in neurotrophin‐related mechanisms in NPH, the causal role of neurotrophin levels in disease pathogenesis remains unexplored using Mendelian randomization (MR). Here, we conducted an MR analysis to evaluate whether neurotrophins exhibit a causal association with NPH susceptibility.

## 2 Materials and Methods

### 2.1 Study Design

**FIGURE 1:**
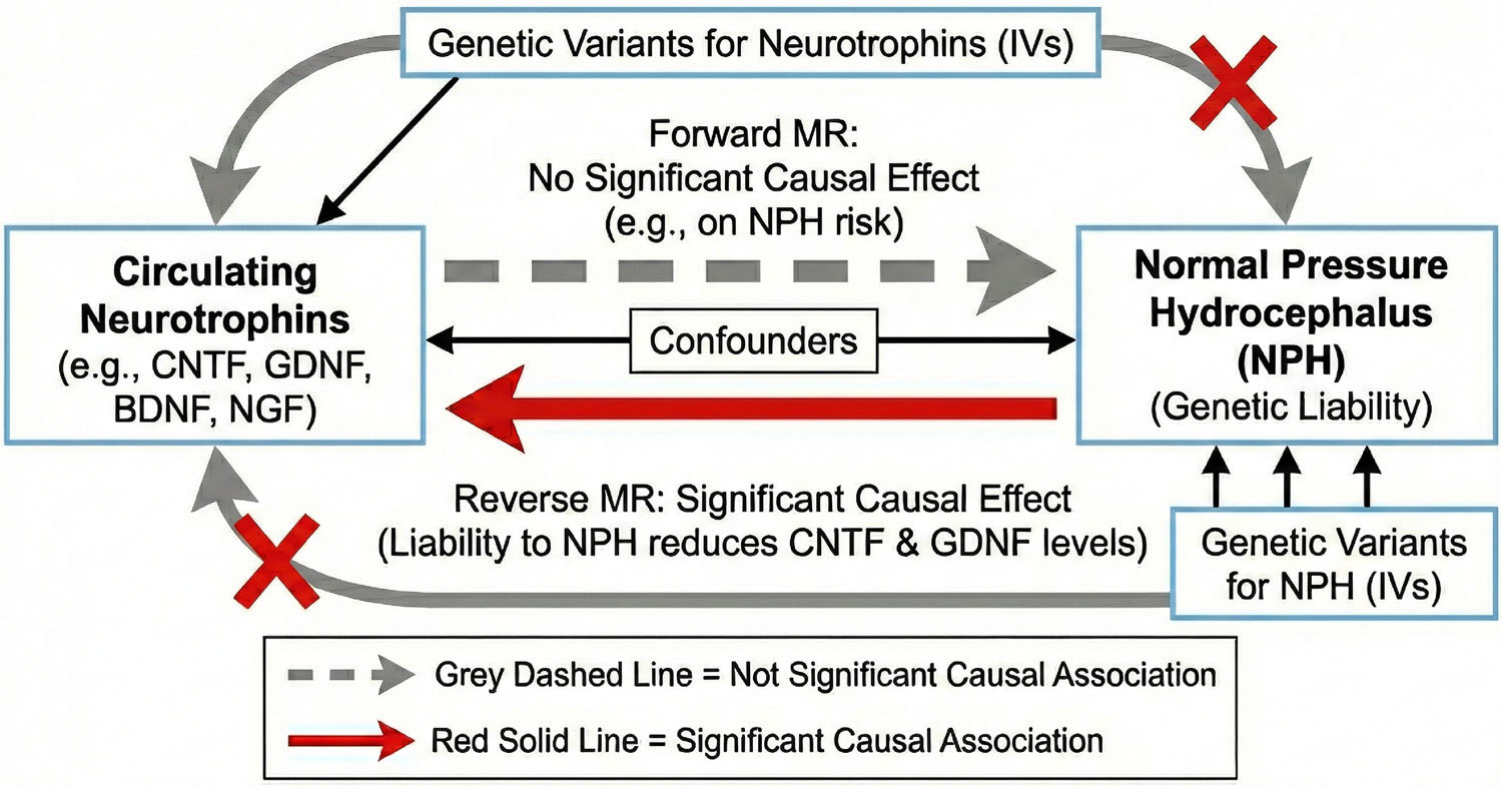
Overview of Mendelian randomization. The red solid arrow indicates a significant causal association, while the grey dashed arrow indicates a non‐significant association.

MR analysis employs genetic variants as IVs in an IV analysis framework (Emdin et al. 2017; Li et al. 2022). The MR Analysis has three important assumptions (Figure 1). Firstly, the genetic variants used as IVs must exhibit a robust and consistent relationship with the exposure of interest. Secondly, these genetic variations should remain independent of any confounding factors, ensuring their purity as proxy measures. Lastly, it is assumed that these selected genetic variants influence the risk of the outcome exclusively through the target risk factors, without any interference from alternate pathways. These foundational principles guide the application and interpretation of MR analysis (Boehm and Zhou 2022).

### 2.2 Data Source

Genome‐wide association study (GWAS) data of BDNF (5,366 cases), NGF (21,758 cases), NT‐3 (33,987 cases), NT‐4 (21,758 cases), CNTF (997 cases), and GDNF (456,348 cases) were obtained from published articles (Gudjonsson et al. 2022; Suhre et al. 2017; Zhao and Li 2023). The GWAS statistics of NPH (1,456 cases and 409,726 controls) were obtained from the FinnGen consortium. All ethnic data originated from European sources. More information about the exposure and outcome datasets is detailed in Table 1.

**TABLE 1:** The GWAS data for exposure and outcomes.

Trait	GWAS ID	Population case/control	Decent
NPH	G6_HCNP	1,456/409,726	European
BDNF	GCST90087740	5,366	European
NGF	GCST90089211	5,365	European
NT‐3	GCST90089211	33,987	European
NT‐4	GCST90274829	21,758	European
CNTF	GCST90100830	997	European
GDNF	GCST90274792	14,736	European

Abbreviations: BDNF, Brain‐derived neurotrophic factor; CNTF, Ciliary neurotrophic factor levels; GDNF, Glial cell line‐derived neurotrophic factor levels; NGF, Nerve growth factor; NPH, Normal pressure hydrocephalus; NT‐3, Neurotrophin‐3; NT‐4, Neurotrophin‐4.

### 2.3 Instrumental Variable (IV) Selection

MR analysis was performed following three main assumptions (Sekula et al. 2016). The first critical step involved screening single nucleotide polymorphisms (SNPs) that were significantly linked to the exposures being investigated. To achieve this, a genome‐wide association study (GWAS) was conducted, focusing on SNPs that exhibited significant associations with neurotrophin levels and NPH, with a statistical significance threshold set at *p* < 5×10^−8^ (Chen et al. 2021). Following the initial screening, the researchers imposed a minor allele frequency (MAF) threshold of 0.01 on the SNPs of interest(Kim et al. 2023). This step aimed to filter out SNPs that were too infrequent in the population, as they may not provide sufficient statistical power for the analysis. Addressing the issue of linkage disequilibrium (LD) among SNPs was the next crucial step. LD, a phenomenon where certain SNPs tend to be inherited together, could introduce bias into the results. Therefore, SNPs with strong LD were excluded from the analysis, using an r^2^ threshold of less than 0.001 and a clumping distance set at 10,000 kb to ensure independence among the remaining SNPs (Clarke et al. 2012). Fourth, if there is no corresponding SNP in the obtained GWAS, the proxy SNP with a higher LD relationship with the missing SNP is selected using the criterion that R^2^ is greater than 0.8 (Wan et al. 2023). To further refine the analysis and exclude potential weak instrumental biases between the instrumental variables (IVs) and exposure factors, the researchers calculated F‐values for each SNP in the IVs. The F‐value assesses the strength of the IVs by quantifying the proportion of exposure variance explained by the SNPs. The formula used was F = R^2^ * (N‐2) / (1‐R^2^), where R^2^ represents the proportion of exposure variance and N is the sample size. The requirement for a significant F‐value was set at >10 (Palmer et al. 2012), ensuring that only strong IVs were included in the final analysis. Through these rigorous steps, the researchers were able to conduct a comprehensive and reliable MR analysis, shedding light on the complex relationships between SNPs and various health outcomes. Their meticulous approach ensures that the results provide valuable insights into the genetic basis of disease and inform future research and clinical practices.

### 2.4 Statistical Analysis

For features involving multiple IVs, this study employed four widely utilized MR methods, with the random‐effects inverse variance weighted (IVW) approach serving as the primary technique (Bowden et al. 2016), MR‐Egger regression (Sekula et al. 2016), the Weighted Median method (WM) (Bowden et al. 2016), and the Weighted Mode method (Hartwig et al. 2017) as supplementary methods. To investigate whether NPH exerts a causal influence on neurotrophin expression, we performed a reverse MR framework using genetic variants linked to NPH as IVs, where NPH was modeled as the exposure and neurotrophins as the outcome of interest. The IVW method consolidates the Wald ratio estimates of causal effects from multiple variants, assuming all IVs are valid (Burgess et al. 2017). The MR‐Egger approach does not rely on nonzero mean pleiotropy but sacrifices statistical power. However, it is less precise and can be influenced by outlier genetic variants (Bowden et al. 2016). The WM offers robust estimates of effective IVs, assigning at least 50% weight (Bowden et al. 2016). Simultaneously, we applied the Weighted Mode method, which estimates the causal effect based on the subset of SNPs with the highest frequency (Hartwig et al. 2017). By grouping SNPs into subsets according to the similarity of their causal effects, this method focuses on the most consistent signals among the genetic variants (Sekula et al. 2016). The implementation of these sophisticated MR techniques was facilitated by the “Two Sample MR” package in R version 4.3.2. This software enabled us to conduct rigorous and reproducible analyses, ensuring the robustness of our results. To enhance the interpretability of our findings, we visualized the MR results using scatter plots and forest plots. These graphical representations allowed us to visually assess the distribution of causal effect estimates across different genetic variants, thus gaining deeper insights into the potential causal relationship between NPH and neurotrophin expression. Collectively, our study underscores the value of leveraging a comprehensive suite of MR methods to dissect complex causal relationships in genetic epidemiology. By employing a multifaceted approach, we were able to gain a more nuanced understanding of the potential causal influences of NPH on neurotrophin expression, paving the way for future research in this crucial area.

### 2.5 Sensitivity Analysis

Horizontal pleiotropy was assessed using the MR‐Egger intercept test (Sekula et al. 2016) and the MR‐PRESSO global test (Verbanck et al. 2018). Any outliers flagged by the MR‐PRESSO global test were excluded, and a robustness analysis was conducted using the leave‐one‐out approach to confirm the findings. To quantify heterogeneity, we employed both the IVW method and MR Egger regression, utilizing Cochran's Q statistic. A Q value exceeding the number of instruments minus one or a significant Q statistic at a *p*‐value < 0.05 suggests the presence of heterogeneity or invalid instruments.

## 3 Results

### 3.1 SNP Selection

We first assessed the causal link between neurotrophins and NPH. When neurotrophins were used as exposure factors, 63 relevant IVs were screened, and the F‐values were all greater than 10; When NPH was designated as the outcome in the MR framework, two SNPs were removed from the analysis because their summary statistics lacked compatibility with the required data fields, and no proxy SNP was found (Table S1).

When NPH was used as the exposure factor for the reverse analysis, seven pertinent instrumental variables (IVs) were carefully identified, with each exhibiting F‐values surpassing 10, indicating strong associations. For the MR analysis where CNTF served as the outcome, three SNPs were omitted owing to mismatched information in the summary‐level datasets, preventing their inclusion in the IV selection. The specific IVs for each exposure are outlined in Table S1.

### 3.2 Causal Relationship of Neurotrophins on NPH

**FIGURE 2:**
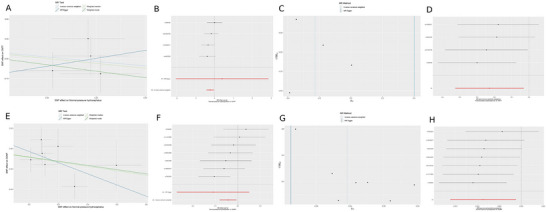
Causal relationship of NPH on CNTF and GDNF. (A): The scatter plot image of CNTF; (B): The forest plot image of CNTF; (C): The funnel plot image of CNTF; (D): The LOO of CNTF; (E): The scatter plot image of GDNF; (F): The forest plot image of GDNF; (G): The funnel plot image of GDNF; (H): The LOO of GDNF.

Genetic prediction studies failed to uncover any statistical link between neurotrophins and NPH. With NPH established as the outcome variable, the application of the IVW method yielded findings wherein none surpassed the conventional significance threshold. Of particular note, BDNF, a historically prominent focus of research interest, exhibited an OR of 1.0679, nestled within a confidence interval ranging from 0.8658 to 1.3163. Table 2, Figure 2. Additionally, three other methods, namely MR‐Egger, WM, and Weighted Mode, also indicated no statistical significance.

**TABLE 2:** Relationship between Neurotrophins and iNPH.

outcome	exposure	N.SNP	Methods	OR_CI	*p*‐value
NPH	NT‐4	9	IVW	0.9576 (0.7687 ‐ 1.1929)	0.699
	NT‐4	9	MR Egger	1.5412 (0.9109 ‐ 2.6076)	0.1509
	NT‐4	9	WM	1.0685 (0.8012 ‐ 1.4249)	0.652
	NT‐4	9	Weighted mode	1.1112 (0.6998 ‐ 1.7645)	0.6668
	NT‐3	10	IVW	0.9615 (0.8476 ‐ 1.0906)	0.5411
	NT‐3	10	MR Egger	0.7784 (0.4808 ‐ 1.26)	0.3377
	NT‐3	10	WM	0.9221 (0.7771 ‐ 1.0943)	0.3532
	NT‐3	10	Weighted mode	0.8569 (0.6507 ‐ 1.1284)	0.2999
	BDNF	8	IVW	1.0679 (0.8663 ‐ 1.3163)	0.5384
	BDNF	8	MR Egger	1.4937 (0.9658 ‐ 2.3103)	0.1214
	BDNF	8	WM	1.1682 (0.8878 ‐ 1.5371)	0.2669
	BDNF	8	Weighted mode	1.2628 (0.9162 ‐ 1.7405)	0.197
	CNTF	24	IVW	0.9658 (0.8783 ‐ 1.062)	0.4725
	CNTF	24	MR Egger	0.8091 (0.5786 ‐ 1.1313)	0.2285
	CNTF	24	WM	1.03 (0.9191 ‐ 1.1542)	0.6115
	CNTF	24	Weighted mode	1.1024 (0.8859 ‐ 1.3718)	0.3911
	NGF	6	IVW	1.2342 (0.9435 ‐ 1.6144)	0.1246
	NGF	6	MR Egger	1.4193 (0.8503 ‐ 2.3691)	0.2514
	NGF	6	WM	1.232 (0.8733 ‐ 1.7382)	0.2347
	NGF	6	Weighted mode	1.2408 (0.7939 ‐ 1.9394)	0.3871
	GDNF	3	IVW	0.9431 (0.6988 ‐ 1.2727)	0.7016
	GDNF	3	MR Egger	0.7194 (0.3685 ‐ 1.4046)	0.5114
	GDNF	3	WM	0.9251 (0.6812 ‐ 1.2564)	0.6182
	GDNF	3	Weighted mode	0.9095 (0.6485 ‐ 1.2754)	0.6375
BDNF	NPH	7	IVW	1.0067 (0.9346 ‐ 1.0844)	0.8597
BDNF		7	MR Egger	1.12 (0.8227 ‐ 1.5247)	0.5037
BDNF		7	WM	0.9889 (0.9111 ‐ 1.0733)	0.7893
BDNF		7	Weighted mode	0.9938 (0.8748 ‐ 1.1291)	0.9272
CNTF		4	IVW	0.7963 (0.6537 ‐ 0.9701)	0.0237
CNTF		4	MR Egger	1.4943 (0.128 ‐ 17.4415)	0.7791
CNTF		4	WM	0.781 (0.6122 ‐ 0.9963)	0.0466
CNTF		4	Weighted mode	0.7218 (0.5118 ‐ 1.0179)	0.16
GDNF		7	IVW	0.9576 (0.9226 ‐ 0.994)	0.023
GDNF		7	MR Egger	0.896 (0.7632 ‐ 1.052)	0.2376
GDNF		7	WM	0.9575 (0.9125 ‐ 1.0048)	0.0776
GDNF		7	Weighted mode	0.9596 (0.8834 ‐ 1.0423)	0.3661
NT‐4		7	IVW	0.9971 (0.9145 ‐ 1.0871)	0.947
NT‐4		7	MR Egger	1.0546 (0.7113 ‐ 1.5637)	0.8019
NT‐4		7	WM	1.009 (0.9107 ‐ 1.118)	0.8638
NT‐4		7	Weighted mode	1.0501 (0.9213 ‐ 1.1969)	0.4915
NGF		7	IVW	1.0632 (0.9913 ‐ 1.1403)	0.0863
NGF		7	MR Egger	1.3332 (1.0575 ‐ 1.6809)	0.0592
NGF		7	WM	1.0622 (0.978 ‐ 1.1536)	0.152
NGF		7	Weighted mode	1.0618 (0.9454 ‐ 1.1927)	0.3504
NT‐3		7	IVW	0.9769 (0.9423 ‐ 1.0127)	0.2025
NT‐3		7	MR Egger	1.1164 (0.9597 ‐ 1.2987)	0.213
NT‐3		7	WM	0.9678 (0.9231 ‐ 1.0146)	0.1737
NT‐3		7	Weighted mode	0.9649 (0.8926 ‐ 1.0431)	0.4037

Abbreviations: BDNF, Brain‐derived neurotrophic factor; CNTF, Ciliary neurotrophic factor levels; GDNF, Glial cell line‐derived neurotrophic factor levels; IVW, Inverse variance weighted; NGF, Nerve growth factor; NPH, Normal pressure hydrocephalus; NT‐3, Neurotrophin‐3; NT‐4, Neurotrophin‐4; WM, Weighted median.

Furthermore, the Cochran's Q statistic (MR‐IVW) confirmed that the MR analyses were robust (Table 3). To investigate the presence of horizontal pleiotropy, we conducted an intercept test within the MR Egger analysis. The results of this test, as detailed in Table 3, indicated no significant horizontal pleiotropy in our studies examining neurotrophins and NPH, with a *p*‐value exceeding 0.05. The MR‐PRESSO results showed statistical significance when NGF was the exposure, but the statistical difference disappeared after correction (Table 4). Lastly, leave‐one‐out analysis underscored the reliability of our causal estimates by affirming that none of the individual SNPs appreciably influenced the associations between neurotrophins and NPH.

**TABLE 3:** Tests for horizontal pleiotropy and heterogeneity.

Outcome	Exposure	Heterogeneity		Pleiotropy	
		Q statistic (IVW)	*p* value	MR‐Egger Intercept	*p* value
NPH	NT‐4	8.783	0.361	−0.094	0.094
	NT‐3	6.6	0.679	0.058	0.399
	BDNF	7.032	0.426	−0.072	0.137
	CNTF	32.873	0.083	0.045	0.292
	NGF	1.561	0.906	−0.027	0.564
	GDNF	0.859	0.651	0.074	0.538
BDNF	NPH	9.529	0.146	−0.031	0.515
CNTF		2.25	0.522	−0.149	0.665
GDNF		6.488	0.371	0.019	0.441
NT‐4		7.485	0.278	−0.016	0.785
NGF		8.615	0.196	−0.066	0.105
NT‐3		5.263	0.511	−0.038	0.135

Abbreviations: BDNF, Brain‐derived neurotrophic factor; CNTF, Ciliary neurotrophic factor levels; GDNF, Glial cell line‐derived neurotrophic factor levels; NGF, Nerve growth factor; NPH, Normal pressure hydrocephalus; NT‐3, Neurotrophin‐3; NT‐4, Neurotrophin‐4.

**TABLE 4:** MR‐Presso results.

Outcome	Exposure	Raw		Outlier corrected		Global *p*	outliers	Distortion *p*
		OR_CI	*P*	OR_CI	*P*			
NPH	BDNF	1.0679 (0.8663 ‐ 1.3163)	0.5579	NA (NA—NA)		0.413	NA	NA
	CNTF	0.9658 (0.8783 ‐ 1.062)	0.4797	NA (NA—NA)		0.087	NA	NA
	GDNF	0.876 (0.6544 ‐ 1.1725)	0.439	NA (NA—NA)		0.574	NA	NA
	NGF	1.2342 (1.0622 ‐ 1.434)	0.0404	NA (NA—NA)		0.908	NA	NA
	NT‐3	0.9615 (0.8631 ‐ 1.0711)	0.4935	NA (NA—NA)		0.679	NA	NA
	NT‐4	0.9576 (0.7687 ‐ 1.1929)	0.7091	NA (NA—NA)		0.391	NA	NA
BDNF	NPH	1.0067 (0.9346 ‐ 1.0844)	0.8655	NA (NA—NA)		0.126	NA	NA
CNTF		0.7963 (0.6712 ‐ 0.9448)	0.0796	NA (NA—NA)		0.558	NA	NA
GDNF		0.9576 (0.9226 ‐ 0.994)	0.0633	NA (NA—NA)		0.362	NA	NA
NGF		1.0632 (0.9913 ‐ 1.1403)	0.1372	NA (NA—NA)		0.222	NA	NA
NT‐3		0.9769 (0.9445 ‐ 1.0104)	0.2224	NA (NA—NA)		0.475	NA	NA
NT‐4		0.9971 (0.9145 ‐ 1.0871)	0.9491	NA (NA—NA)		0.286	NA	NA

Abbreviations: BDNF, Brain‐derived neurotrophic factor; NGF, Nerve growth factor; NT‐3, Neurotrophin‐3; NT‐4, Neurotrophin‐4; GDNF, Glial cell line‐derived neurotrophic factor levels; CNTF, Ciliary neurotrophic factor levels; NPH, Normal pressure hydrocephalus.

### 3.3 Causal Relationship of NPH on Neurotrophins

When CNTF was designated as the outcome variable in the MR framework, the IVW estimator indicated a significant protective effect (OR = 0.7963, 95% CI: 0.6537–0.9701), achieving statistical significance (*p* = 0.0237). Similarly, the WM method showed that NPH had a negatively causal relationship with CNTF (OR (95% CI): 0.7810 (0.6211‐0.9964), *p* = 0.0466). When GDNF served as the outcome variable, the IVW method showed a non‐significant trend towards a protective effect, with an OR (95% CI) of 0.9576 (0.9226‐0.9940), although it achieved statistical significance at *p* = 0.0230. However, this casual association was not confirmed by other methods. Notably, the MR‐Egger, WM, and Weighted Mode methods did not yield statistically significant results for any of the outcomes (Table 2, Figure 2).

In terms of sensitivity analysis, MR‐Egger regression highlighted the influence of pleiotropy in relation to NPH, and the findings indicated that pleiotropy did not introduce bias into the MR analysis, suggesting robustness of the causal estimates. (*p* > 0.05) (Table 3). Statistical assessment via Cochran's Q test and visual inspection of funnel plots revealed minimal heterogeneity across IVs for neurotrophins, thereby validating the robustness of the IVW estimates (Table 3). MR‐PRESSO analysis identified no outliers, and the leave‐one‐out test demonstrated the stability of the MR results, attesting to their robustness (Table 4).

## 4 Discussion

Mendelian randomization (MR) capitalizes on genetic diversity to delve into risk factors and disease consequences. Our research uses a bidirectional MR framework to explore the causal association between neurotrophins and NPH. We provide evidence that genetically, NPH reduces the levels of CNTF and GDNF. Sensitivity analyses confirmed that these outcomes were robust. This discovery hints at an intricate interplay between NPH and these neurotrophic factors, offering fresh insights into the pathophysiological mechanisms underlying neurological disorders.

Both CNTF and GDNF belong to the family of neurotrophic factors, which are involved in the process of neuronal survival, differentiation, and regeneration (Dulz et al. 2020; Kimura et al. 2016). These factors are vital for maintaining the balanced functioning of the nervous system and initiating repair mechanisms in response to injuries (Kimura et al. 2016; Savolainen et al. 2018). Studies have shown that patients with major depression and Alzheimer's disease exhibit lower levels of plasma GDNF compared to healthy individuals, suggesting its potential involvement in these disorders (Straten et al. 2009; Sun et al. 2019). Furthermore, GDNF has demonstrated efficacy in treating Parkinson's disease (Kakoty et al. 2024). Similarly, CNTF was strongly associated with neurological disorders (Guo et al. 2022). We found that NPH may be a negative factor for neurotrophic factor CNTF levels and GDNF levels, which means that the occurrence of NPH may decrease the levels of the above mentioned neurotrophic factors. This may explain the neurological complications in NPH patients.

However, MR estimates reflect lifelong genetic liability rather than acute disease status. The genetic predisposition to NPH likely induces subtle, chronic alterations in cerebrospinal fluid (CSF) dynamics and intracranial compliance. We hypothesize that such long‐term exposure to microenvironmental stress—including sustained hemodynamic changes and low‐grade inflammation—could cumulatively impair the metabolic support for neurons and glial cells (Czosnyka and Czosnyka 2017; Evensen and Eide 2020; Xiang et al. 2023). Consequently, this chronic physiological burden may gradually downregulate the synthesis of neurotrophic factors (e.g., CNTF and GDNF) throughout the lifespan, rather than solely acting as an acute complication (Bao et al. 2024).

Furthermore, established NPH pathology is often accompanied by significant inflammatory responses and oxidative stress (Czubowicz et al. 2017; Guzelcicek et al. 2021), processes that directly damage neurons and supporting cells, further reducing neurotrophin production and exacerbating neurological damage. In addition, physical compression and structural distortions caused by NPH may interfere with axonal transport (Weller 1998), hindering the delivery of neurotrophic factors from the cell body to nerve terminals, resulting in decreased local concentrations.

From a molecular perspective, the concurrent downregulation of CNTF and GDNF implies shared vulnerability in their regulatory pathways. Both factors are primarily synthesized by astrocytes and are regulated by the JAK/STAT signaling cascade [PMID:10812968]. While acute astrogliosis typically upregulates these factors, the chronic pathological stress inherent to NPH can disrupt JAK/STAT signaling efficiency or lead to ‘glial exhaustion’ (Eide 2022), thereby underpinning the parallel decline of both neurotrophins.

From a clinical perspective, these findings underscore the potential of neuroprotective strategies. Firstly, exogenous supplementation of neurotrophic factors could serve as a direct neuroprotective measure. Emerging therapies delivering CNTF or GDNF have shown potential in mitigating neuronal degeneration in other neurological conditions (Thoenen and Sendtner 2002), suggesting a similar applicability for shielding injured neurons in NPH. Secondly, and perhaps more distinctively for NPH, is the restoration of the endogenous neuro‐glial microenvironment. Evidence suggests that effective cerebrospinal fluid shunting not only alleviates physical compression but may also reduce inflammatory metabolites (Sosvorova et al. 2015), potentially restoring the brain's intrinsic capacity to synthesize neurotrophins. Although the odds ratios observed in our study suggest a modest reduction quantitatively, the biological impact should not be underestimated. Given that neurotrophins function as potent signaling molecules even at low concentrations, such persistent, lifelong downregulation represents a cumulative deficit that likely compromises the neuronal maintenance threshold over decades (Chao 2003).

Our research has also delved into the complex relationship between other neurotrophins, including NT‐3/4, BDNF, and NGF, and NPH. While clinical studies have consistently shown an association between these neurotrophins and NPH, our findings challenge the notion of a direct causal link. Specifically, our forward MR analysis indicated no significant causal effect of genetically predicted levels of these neurotrophins on NPH risk. This null finding is biologically plausible. NPH etiology is predominantly driven by biomechanical and hemodynamic factors—such as impaired cerebrospinal fluid reabsorption and reduced intracranial compliance—which are not directly regulated by neurotrophic factors (Mani et al. 2024). In contrast, earlier case‐control studies observed that levels of NT‐3 and NGF were elevated in the cerebrospinal fluid of hydrocephalus patients (Hochhaus et al. 2001), while others reported significantly lower serum BDNF concentrations in NPH patients compared to controls (Laske et al. 2007; Mehmedika‐Suljić et al. 2023). We propose that these clinical correlations are likely attributable to reverse causation or confounding rather than a direct forward effect. As supported by our reverse MR results, the established NPH disease state may lead to secondary alterations in neurotrophic support, rather than the neurotrophins initiating the disease. By leveraging genetic variants as naturally occurring instruments, our MR study minimizes the confounding bias often present in observational studies (e.g., age, comorbidities), thereby providing robust evidence against a primary causal link between these neurotrophin levels and NPH risk (Holmes et. 2017). These findings suggest that neurotrophin levels alone are typically downstream markers rather than upstream risk factors for NPH, a consideration that warrants integration into future clinical research designs.

Our Mendelian randomization (MR) study offers several methodological strengths. First, genetic variants serve as stable and directly measurable instruments, minimizing confounding bias due to their fixed nature at conception and independence from environmental influences. Second, the analysis incorporated a broad panel of neurotrophic factors, a large‐scale dataset, and a bidirectional MR framework with no evidence of pleiotropy or heterogeneity, enhancing the reliability of the causal inferences. Third, the genetic instruments utilized in this study—derived from genome‐wide association study summary statistics—are randomly allocated prenatally, thereby acting as unconfounded proxies for modifiable exposures, analogous to the randomization principle in RCTs within observational settings. Finally, the two‐sample MR design leveraged a substantial sample size, bolstering statistical power and confidence in the findings. However, despite the robustness of two‐way MR for causal inference, potential limitations warrant attention, such as the reliance on European‐ancestry populations, which may limit generalizability. To address these concerns and further validate our findings, follow‐up studies are essential. Repeating experiments in diverse cohorts and utilizing multiple MR analysis methods will provide a more comprehensive assessment of our conclusions. Additionally, incorporating animal models and cellular experiments will offer deeper insights into the underlying biological mechanisms driving the observed associations. Finally, it should be noted that MR estimates reflect the effects of lifelong genetic exposure, which may differ from the short‐term effects of clinical disease progression or therapeutic interventions. While our findings suggest that genetic liability to NPH is causally associated with reduced neurotrophin levels, we cannot strictly distinguish whether this reduction is driven by early developmental changes or accumulated pathological stress in later life. By addressing these limitations and validating our findings through diverse methodologies, we aim to contribute to a more robust and generalizable understanding of the complex interactions between genetic factors and phenotypic outcomes.

## 5 Conclusion

The MR analysis indicated that NPH negatively influences neurotrophic factors, specifically reducing levels of CNTF and GDNF. These findings enhance our comprehension of the intricate relationship between NPH and neurotrophic factors. Furthermore, they offer a pivotal theoretical foundation and research trajectory for exploring innovative treatment pathways for neurological disorders. Future research should delve deeper into the precise mechanism of this interaction and its potential translation into effective clinical strategies for treating such diseases.

## Author Contributions

Tao Xu and Qiang Gan carried out the studies, participated in collecting data, and drafted the manuscript. Tao Xu and Handong Wang performed the statistical analysis and participated in its design. Tao Xu, Haifeng Liu, and Xiwen Huang participated in the acquisition, analysis, or interpretation of data and drafted the manuscript. All authors read and approved the final manuscript.

## Funding

This work was supported by the National Natural Science Foundation project (No: 82271426).

## Conflicts of Interest

The authors declare no conflicts of interest.

## Supporting information




**Supplementary Materials**: brb371309‐sup‐0001‐TableS1.xlsx

## Data Availability

All data generated or analyzed during this study are included in this published article.
